# The Prognostic Value of Cardiac Troponin I in Patients with or without Three-Vessel Disease Undergoing Complete Percutaneous Coronary Intervention

**DOI:** 10.3390/jcm11133896

**Published:** 2022-07-04

**Authors:** Zhi-Fan Li, Shuang Zhang, Hui-Wei Shi, Wen-Jia Zhang, Yong-Gang Sui, Jian-Jun Li, Ke-Fei Dou, Jie Qian, Na-Qiong Wu

**Affiliations:** Cardiometabolic Center, National Center for Cardiovascular Diseases, Fuwai Hospital, Chinese Academy of Medical Science & Peking Union Medical College, Beijing 100037, China; lizajackson@foxmail.com (Z.-F.L.); tats246@163.com (S.Z.); 15770738175@163.com (H.-W.S.); wendyzhang0325@163.com (W.-J.Z.); fwsyg999@163.com (Y.-G.S.); lijianjun938@126.com (J.-J.L.); drdoukefei@126.com (K.-F.D.); qianjfw@163.com (J.Q.)

**Keywords:** percutaneous coronary intervention, cardiac troponin, three-vessel disease, prediction

## Abstract

Postprocedural cardiac troponin I (cTnI) elevation commonly occurs in patients undergoing percutaneous coronary intervention (PCI); however, its prognostic value remains controversial. This study aimed to investigate the prognostic value of peak postprocedural cTnI in cardiac patients with or without three-vessel disease (TVD) undergoing complete PCI. A total of 1237 consecutive patients (77% males, mean age 58 ± 10 years) with normal baseline cTnI levels were enrolled, 439 patients (77% males, 59 ± 10 years) with TVD, and 798 patients (77% males, 57 ± 10 years) with single- or double-vessel disease (non-TVD). The primary outcome was the occurrence of major adverse cardiovascular events (MACE), defined as a composite of non-fatal MI, non-fatal stroke, unplanned revascularization, re-hospitalization due to heart failure or severe arrhythmias, and all-cause death. During the median follow-up of 5.3 years, a total of 169 patients (13.7%) developed MACE, including 73 (16.6%) in the TVD group and 96 (12.0%) in the non-TVD group (*p* = 0.024). After adjustment, the multivariate Cox analysis showed that hypertension (HR 1.50; 95% CI: 1.01–2.20; *p* = 0.042), TVD (HR 1.44; 95% CI: 1.03–2.02; *p* = 0.033), and cTnI ≥ 70× URL (HR 2.47; 95% CI: 1.28–4.78, *p* = 0.007) were independently associated with increased MACE during long-term follow-up. Further subgroup analyses showed that cTnI ≥ 70× URL was an independent predictor of MACE in TVD patients (HR 3.32, 95% CI: 1.51–7.34, *p* = 0.003), but not in non-TVD patients (HR 1.01, 95%CI: 0.24–4.32, *p* = 0.991). In conclusion, elevation of post-PCI cTnI ≥ 70× URL is independently associated with a high risk of MACE during long-term follow-up in patients with TVD, but not in those with non-TVD.

## 1. Introduction

Cardiac troponin I (cTnI) is a sensitive marker of myocardial damage and necrosis, and is expressed only in myocardium [1]. Postprocedural cTnI elevation commonly occurs in patients with coronary heart disease (CAD) undergoing percutaneous coronary intervention (PCI) and may significantly relate to poor outcomes [2,3]. Current expert consensus states that elevated postoperative troponin is an important marker of procedure-related myocardial injury, even procedure-related myocardial infarction (MI), and different thresholds of troponin elevation define the degree of injury [4,5,6,7]. However, there is controversy over the predictive value of cTnI for the prognosis of patients undergoing PCI. Several studies have indicated that postprocedural cTn elevation is valuable for predicting of poor outcomes [8,9,10,11,12,13], whereas other studies have shown that it has no impact on prognosis even if the elevation is extremely high [14,15].

Three-vessel coronary artery disease (TVD) is a severe and complex type of CAD, defined as a lesion of >50% diameter stenosis (DS) in all three main epicardial coronary arteries, including the left anterior descending artery (LAD), the left circumflex artery (LCX), and the right coronary artery (RCA), with or without left main artery (LM) involvement. Patients with TVD have a higher risk of death and major adverse cardiac events (MACEs) [16,17,18]. In the SYNTAX (Synergy Between PCI With Taxus and Cardiac Surgery) trial, involving patients with TVD or LM, PCI-related myocardial damage was associated with all-cause mortality. However, this study only used creatine kinase–myocardial band (CK-MB) as a marker because cTnI was not collected [19]. More recently, studies on cTnI and post-PCI prognosis have been conducted in patients with acute myocardial infarction (AMI) [20], LM disease [21], chronic total occlusion (CTO) [22], and diabetes mellitus (DM) [23] who are undergoing PCI, but there is a lack of studies involving patients with TVD.

Here, our study focused on the predictive value of postprocedural cTnI for the long-term prognosis of patients undergoing complete PCI. Furthermore, our study explored variance in the prognostic performance of postprocedural cTnI between TVD and non-TVD patients and assessed the impact of different cTnI levels on outcomes in TVD patients.

## 2. Materials and Methods

### 2.1. Study Participants

We included consecutive CAD patients with normal preoperative cTnI levels who successfully underwent complete PCI at the Department of Cardiology, Fuwai Hospital, Chinese Academy of Medical Science between January 2011 and December 2013, and who consented for long-term follow-up. The inclusion criteria were as follows: (1) age <75 years; (2) acute coronary syndrome (ACS) or stable CAD with an indication for PCI; and (3) undergoing complete revascularization (CR). CR was defined by a combination of the operator’s judgments of a patient’s coronary angiography lesions and clinical manifestations (some patients had positive results in non-invasive examinations). TVD-CR was defined by the presence of >50% DS in the three coronary arteries (LAD, LCX, RCA), and the operator completed the revascularization treatment of lesions with interventional indications (i.e., segments >2.0 mm in diameter with ≥75% DS). The exclusion criteria were as follows: (1) the absence of postoperative cTnI measurements; (2) coronary stenosis with <50% DS in the main epicardial coronary arteries; (3) the presence of LM stenosis with >50% DS; (4) the presence of chronic total occlusion (CTO); (5) severe liver and kidney dysfunction; (6) combined cardiomyopathy or malignancies; (7) a left ventricular ejection fraction (LVEF) of <35%; (8) previous aorto-coronary bypass grafting surgery (CABG); and (9) the presence of valvular heart disease.

Baseline patient demographics, clinical and laboratory examinations, procedural characteristics, and medication at discharge were prospectively collected in a designed database by independent researchers. Risk factors, such as diabetes and hypertension, were determined based on medical record system queries or questionnaires. An echocardiogram was performed at the time of admission. This study was approved by the Fuwai Hospital Ethics Committee and was conducted in accordance with the Declaration of Helsinki. All participants provided written informed consent.

### 2.2. Procedural and Biomarker Assessment

PCI was performed by experienced interventionists according to standard clinical practices. The choice of PCI technique and stent type was at the discretion of the operators. Prior to PCI procedures, dual antiplatelet therapy (DAPT) was administered and continued for at least one year, according to AHA/ACC and ESC recommendations at the time of the index procedure [24,25,26,27]. Sufficient doses of dual antiplatelet medicines were administered before PCI procedures, and thereafter dual antiplatelet medicines were administered for at least 1 year.

Blood samples were routinely collected for cTnI testing before angiography (baseline cTnI level), within 8–24 h after PCI, and every morning thereafter during hospitalization. The patients’ plasmid lipid profile, hemoglobin, and N-terminal pro-brain natriuretic peptide (NT-proBNP) levels were analyzed at the central chemistry laboratory. Standard cTnI levels were detected using a commercial chemiluminescence method through the Beckman Coulter (Brea, CA, USA) access immunoassay system and were normalized to the upper reference limit (URL). Patients with cTnI under 0.04 ng/mL (99th centile URL) were assessed as normal, as determined by the manufactures.

### 2.3. Follow-Up and Clinical Outcomes

Clinical follow-up information was collected from the patients’ medical records on subsequent visits and from routine telephone or message-based surveys by research staff who were blinded to this study. All events were adjudicated by independent medical personnel who were not involved in the PCI procedure. The endpoint of interest was the occurrence of major adverse cardiovascular events (MACEs), defined as a composite of non-fatal MI, non-fatal stroke, unplanned revascularization, re-hospitalization due to heart failure or severe arrhythmias, and all-cause death.

### 2.4. Statistical Methods

Statistical analyses were performed using R version 4.1.1 (R Foundation for Statistical Computing, Vienna, Austria). The Shapiro–Wilk test was used to examine the normality of continuous variables. Data are reported as mean ± standard deviation (X ± SD) or median (interquartile range) for normal and non-normally distributed continuous variables, and as counts with percentages [n, (%)] for categorical variables. Differences between continuous variables were analyzed using the Student’s unpaired t-test or Wilcoxon rank sum tests as appropriate, whereas differences between categorical variables were analyzed using the Chi-squared or Fischer’s exact test depending on the size of the group.

The cumulative event-free survival data are presented graphically using the Kaplan–Meier method, and the differences among prespecified commonly used cTnI interval groups (<1×, 1–5×, 5–69×, and ≥70× URL) were compared using log-rank tests. Univariate and multivariate Cox regression analyses were used to investigate the impacts of the variables on long-term MACEs after PCI, with hazard ratios (HRs) and 95% confidence intervals (CIs). Age, sex, hypertension, DM, systolic blood pressure, LVEF, hemoglobin, and NT-proBNP were included in the adjusted models. Furthermore, the risk of MACEs in response to high cTnI levels and TVD and their interactions were tested. Similarly, Cox proportional hazard models were performed for TVD and non-TVD groups in order to assess the prognostic value of cTnI. All probability values were two-sided, and *p* < 0.05 was considered statistically significant.

## 3. Results

### 3.1. Study Population

A total of 1237 patients (77% males, mean age 58 ± 10 years) with normal preoperative cTnI levels were included in the study. Patients’ baseline clinical characteristics, past history and comorbidities, laboratory results, and medication at discharge are shown in Table 1, and are stratified according to the presence or absence of TVD. Among these patients, 439 (35.5%) were diagnosed with TVD, 348 (28.1%) had single-vessel disease and 450 (36.3%) had double-vessel disease.

Compared to the non-TVD group, the TVD group were older (57 ± 10 vs. 59 ± 10 years, *p* = 0.005), had a higher proportion of patients with hypertension (61.6 vs. 68.2%, *p* = 0.021) and diabetes (25.5 vs. 33.8%, *p* = 0.002), and were more often discharged on beta-blockers (84.4 vs. 88.6%, *p* = 0.044). Furthermore, the TVD group had a significantly higher level of NT-proBNP and a significantly lower level of hemoglobin compared to the non-TVD group (555.8 [448.9706.7] vs. 579.75 [454.58,777.82] pg/mL, *p* = 0.049; 141.22 ± 14.37 vs. 143.09 ± 14.52 g/L, *p* = 0.031, respectively).

cTnI elevation above the 1× URL occurred after 59.7% (738/1237) of the total number of procedures. In comparison with the non-TVD group, the TVD group had a higher proportion of 1× URL ≤ cTnI < 5× URL (31.0 vs. 33.0%), 5× URL ≤ cTnI < 70× URL (23.7 vs. 27.1%), and cTnI ≥ 70× URL (2.5 vs. 4.1%), and a lower proportion of cTnI < 1× URL (42.9 vs. 35.8%). However, the *p*-value did not reach significance (*p* = 0.055).

### 3.2. Outcome Analysis

A total of 169 (13.7%) patients developed MACEs after a median follow-up of 5.3 (4.5–5.5) years. The TVD group developed a significantly higher proportion of MACEs (16.6 vs. 12.0%, *p* = 0.024) and all-cause death (2.7 vs. 1.0%, *p* = 0.021) compared to the non-TVD group.

**Figure 1:**
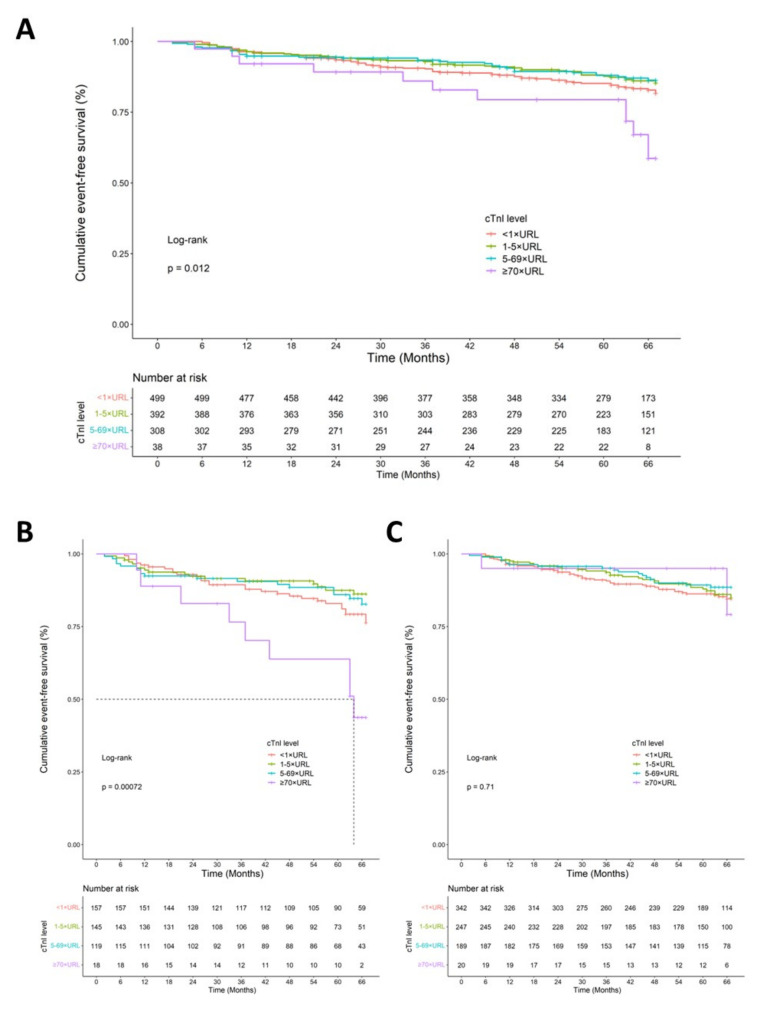
Cumulative Kaplan–Meier survival curve analysis of 5-year MACEs in total patients (**A**), TVD patients (**B**), and non-TVD patients (**C**) according to postprocedural cTnI levels. Notes: MACEs, major adverse cardiovascular events; cTnI, cardiac troponin I; URL, upper reference limit; TVD, three-vessel disease; non-TVD, non-three-vessel disease, including single-vessel disease and double-vessel disease.

The outcomes of different postoperative peak cTnI levels were explored. Patients with normal cTnI were considered the control group, whereas patients with elevated cTnI were divided into three groups (1× URL ≤ cTnI < 5× URL, 5× URL ≤ cTnI < 70× URL, and cTnI ≥ 70× URL). The relationships between peak postprocedural cTnI levels and MACEs are shown in Figure 1A. The Kaplan–Meier analysis showed that outcomes differed according to peak postprocedural cTnI levels, and that cTnI ≥ 70× URL was associated with 5-year MACEs (log-rank test *p* = 0.012). Univariate analyses between common clinical variables and MACEs were performed for the total patient cohort (Table 2). We found that the following variables had significant univariate associations: age (HR 1.02 [1.01,1.04], *p* = 0.005), hypertension (HR 1.56 [1.11,2.19], *p* = 0.01), diabetes mellitus (HR 1.55 [1.14,2.12], *p* = 0.006), TVD (HR 1.42 [1.04,1.92], *p* = 0.026), NT-proBNP (HR 1.00 [1.00,1.001], *p* = 0.008), and cTnI ≥ 70× URL (HR 2.09 [1.11–3.94], *p* = 0.022). After adjusting for all of the variables above, Cox multivariate analyses showed that hypertension (HR 1.50 [1.01–2.20], *p* = 0.042), TVD (HR 1.44 [1.03–2.02], *p* = 0.033), and cTnI ≥ 70× URL (HR 2.47 [1.28–4.78], *p* = 0.007) were associated with increased MACEs. We further divided the total population according to TVD and cTnI levels (70× URL) in Table 3. Among the four groups, patients with TVD and cTnI ≥ 70× URL had the highest risk of experiencing MACEs (50.0%). In the TVD group, those with cTnI ≥ 70× URL had an increased absolute and relative risk of MACEs compared to those with cTnI < 70× URL (HR 4.43, 95% CI: 2.13 to 9.24, *p* < 0.001). In contrast, the MACE risk difference in the non-TVD group was modest (HR 0.78, 95% CI: 0.19 to 3.20, *p* = 0.731). Interaction tests between TVD and the postprocedural peak cTnI level on the 5-year risk of MACE reached the significant cutoff value (*p* = 0.05). A sensitivity analysis for the interaction between TVD and cTnI ≥ 70× URL was performed according to clinical setting (ACS vs non-ACS). In the ACS subgroup (Supplementary Table S1), TVD patients with cTnI ≥ 70× URL were independently associated with a higher risk of MACE (HR: 5.30, 95% CI: 2.29–12.25, *p* < 0.001) (*p* for interaction = 0.04). However, in the non-ACS subgroup, the number of events was insufficient to analyze (Supplementary Table S2).

We further analyzed prognosis for different cTnI thresholds among TVD and non-TVD patients. In the TVD cohort, the Kaplan–Meier survival curve showed significantly lower event-free survival in patients with cTnI ≥ 70× URL compared to patients with normal postoperative peak cTnI (<1× URL) (log-rank *p* < 0.001) (Figure 1B). The log-rank test showed no significant difference for patients with 5× URL ≤ cTnI < 70× URL (*p* = 0.4) and 1× URL ≤ cTnI < 5× URL (*p* = 0.1). The multivariate Cox regression analysis, adjustedfor the covariates discussed above, showed that only cTnI ≥ 70× URL was associated with a significantly higher rate of MACE during long-term follow-up when compared with the reference cTnI value (HR 3.32; 95%CI: 1.51–7.34; *p* = 0.003) (Table 4). However, such an association was not present for non-TVD patients with any range of post-PCI peak cTnI (Figure 1C). The Cox regression analysis for a single adverse event showed that unplanned revascularization had a significantly higher HR in the TVD group with cTnI ≥ 70× URL (HR 4.94, 95%CI: 1.83–13.30, *p* = 0.002) compared to the reference group (Supplementary Table S3).

## 4. Discussion

To the best of our knowledge, there have been no previous studies exploring the prognostic value of elevated cTnI in a population of TVD patients undergoing complete PCI. This is the first study unveiling the potential prognostic differences of post-PCI cTnI elevation in patients with and without TVD. In this study examining the predictive value of post-PCI cTnI for long-term prognosis in patients with or without TVD, the major findings were: (1) significant elevations in post-PCI peak cTnI levels (cTnI ≥ 70× URL) were independently associated with an increased risk of long-term MACEs in patients with TVD, but not in those with non-TVD; and (2) elevations in post-PCI peak cTnI < 70× URL were not prognostically significant.

Cardiac troponin is widely used in the diagnosis of myocardial injury and is significantly associated with increased MACEs and mortality [5,28,29]. The routine assessment of cardiac biomarkers 8 to 12 h after PCI is advised, according to the American College of Cardiology/American Heart Association (ACC/AHA) guidelines for PCI (class II-b recommendation) [26]. Troponin I elevation frequently occurs in patients undergoing PCI [14,30]. In our study, the overall percentage of cTnI elevation was 59.7%, and the incidence was higher in TVD patients (64.2%). As a sensitive and specific biomarker reflecting myocardial injury, assessment of cTnI has been commonly recommended to define procedure-related myocardial injury and periprocedural MI (PMI). The 3rd Universal Definition of Myocardial Infarction (UDMI) identifies cTn as the biomarker used to define PCI-related MI (type 4a MI), and CK-MB is used only if cTn is unavailable [4]. The 4th UDMI defines PMI only by troponin levels [5]. Myocardial injury is defined by the stand-alone detection of postprocedural cTn elevation above the 99th percentile URL [4,5]. The European Association of Percutaneous Cardiovascular Interventions (EAPCI) proposed that a >5-fold increase in post-PCI cTn values is a prognostically relevant “major” periprocedural myocardial injury [6]. The definition of type 4a MI is not only based on the same cutoff threshold (cTn > 5× URL), but also requires at least one clinical correlate, including ischemic electrocardiogram changes, flow-limiting angiographic complications, and/or supporting evidence obtained from imaging [5,6]. In contrast, groups of interventional cardiologists from the Society of Cardiovascular Angiography and Interventions (SCAI) strongly preferred CK-MB > 10 times the upper limit of normal (ULN, similar to URL) as a PMI threshold, otherwise cTn > 70× ULN. Based on SCAI, the Academic Research Consortium-2 (ARC-2) recommended that post-PCI MI be defined as cTn > 35× ULN with ancillary criteria or cTn > 70× ULN for isolated biomarker elevation [7]. Therefore, in this study, we set threshold intervals of isolated cTnI elevation (<1× URL, 1–5× URL, 5–70× URL, ≥70× URL) based on expert recommendations for myocardial injury, major perioperative myocardial injury, and PMI. 

Previous research exploring the relationship between post-PCI troponin elevation and clinical prognosis has produced inconsistent results [14,31,32]. In our study, we found that 13.7% of patients developed MACEs after a median follow-up of 5.3 years. Furthermore, we found a positive association between large cTnI elevation and MACEs. This result is in accordance with a meta-analysis conducted by Nienhuis et al., who found that post-PCI troponin elevation was significantly associated with increased mortality (4.4% vs. 3.3%, *p* = 0.001; OR 1.35) and incidence of death or nonfatal MI (8.1% vs. 5.2%, *p* < 0.001; OR 1.59) [31]. A recent meta-analysis that pooled all prospective trials (11 studies, 13,932 patients) indicated that elevated cTnI after elective PCI was associated with an increase in all-cause mortality compared with non-elevated cTnI (OR 1.42, 95% CI 1.19 to 1.69, *p* < 0.001), and for every 3 × 99th percentile URL increment of cTnI, the pooled risk of death increased by 33% (95% CI 1.15 to 1.53, *p* < 0.001) [32]. In this study, we used the experts’ suggested cutoff values for the prognostic significance of post-PCI cTnI elevation. As shown in the Kaplan–Meier survival curves, the event-free survival rate was apparently lower when the post-PCI cTnI ratio was no less than 70× URL. Similarly, univariate and multivariate Cox analyses indicated that patients with cTnI ≥ 70× URL were at a significantly higher risk of experiencing MACEs. For patients with normal cTnI (<99th percentile URL), post-PCI 1× URL ≤ cTnI < 5× URL, and 5× URL ≤ cTnI < 70× URL, we found no difference in event-free survival among them. In this regard, our study is consistent with a recent pooled analysis of 4362 elective PCI patients with normal baseline conventional cTn levels. This pooled analysis found a significant association between subsequent 1-year all-cause mortality and post-PCI cTn ≥ 70× URL (adjusted OR, 5.97 95% CI 1.65 to 21.59, *p* = 0.023). Among a total of 9081 patients (including patients with hs-cTn data), 1-year all-cause mortality was strongly associated with the lesser threshold of post-PCI cTn elevation (either hs-cTn or conventional cTn ≥ 5× URL) [33]. Another pooled analysis of 13,452 stable CAD patients undergoing elective PCI compared 1-year mortality in patients with different cTn cutoff values (normal, 1 ≤ cTn < 3, 3 ≤ cTn < 5, 5 ≤ cTn < 10, 10 ≤ cTn < 20, 20 ≤ cTn < 35, 35 ≤ cTn < 70, and ≥70 times ULN). Although cTn ≥ 70× ULN was associated with increased risk of mortality as indicated by the Kaplan–Meier curves, the Cox multivariate analysis showed that the mortality rate did not increase, irrespective of any level of cTn elevation [14]. Discordance may be due to the use of different models for multivariate analysis, a wider primary end-point and a longer follow-up time in our study, and variance in the population. Of note, most patients included in this pooled analysis had single-vessel disease (78%), whereas only 28.2% of the patients in our study had single-vessel disease. 

TVD confers higher rates of mortality and an increased risk of MACEs compared with non-TVD, and is regarded as a severe condition [34,35]. A meta-analysis showed that complete revascularization for TVD significantly reduced the risk of all-cause mortality compared to incomplete revascularization [36]. In this study, all patients underwent complete PCI for clinical benefit, and 439 patients (35.5%) were diagnosed with TVD. A study by Tsai et al. reported that TVD was an independent predictor for the time-dependent occurrence of MACEs in patients with elective PCI after a mean follow-up period of 32 months. The researchers believed that multifactorial occurrence—including TVD—facilitated the association of stent implantation with MACEs [37]. Similarly, the results from our multivariate Cox analysis also showed that TVD was associated with a higher risk of MACEs. Previous studies have reported that the number of target vessels in PCI play a crucial role in the elevation of cardiac enzymes, and multi-vessel PCI could increase the risk of sustained periprocedural myocardial injury or PMI [9,38]. In our population, TVD patients had a higher proportion of post-PCI cTnI elevation. Moreover, cTnI ≥ 70× URL was also an independent predictor of MACEs. Therefore, we further tested and confirmed the interaction between TVD and a high post-PCI cTnI cutoff (70× URL). We suggest that the effect of TVD should be carefully considered in future prognostic studies of cardiac biomarkers.

Postprocedural cardiac enzyme elevation was frequently detected after complex PCI (i.e., CTO, LM, or multivessel PCI), whereas the prognostic impact of cTn remained controversial [13,39]. In this study, we included TVD and non-TVD patients, and found that postoperative cTnI elevation is an independent prognostic value for long-term prognosis only in TVD patients as these patients had a significantly higher occurrence of MACEs when their cTnI levels surpass 70× URL. On the other hand, for non-TVD patients or for those with mild cTnI elevation, our study suggests that there are no significant adverse effects on long-term prognosis. Since delayed-enhancement MRI verified the degree of post-PCI cTnI elevation that correlated directly with the extent of new irreversible myocardial injury [40], patients with higher cTnI elevation were presumed to have more severe perioperative myocardial necrosis. Thus, we speculated that a large range of PCI-induced myocardial injury indicated by cTnI ≥ 70× URL, rather than minor necrosis, would produce a poor prognosis, according to our results. Therefore, the routine measurement of cTnI may be helpful for risk stratification and for the postprocedural management of patients undergoing PCI. Increased awareness of the prognostic value of cTn may contribute to the early detection of high-risk patients and the prevention of postoperative adverse events. Further large-scale prospective trials are needed to confirm the contribution of post-PCI elevated cTnI to prognosis in different patients with more clinically relevant variables.

## 5. Limitations

The present study has several potential limitations. Firstly, we used a single-center, observational study design which included a relatively low rate of MACEs. This may limit the power of our analysis and the generalizability of our results. Secondly, although we attempted to adjust for multiple covariates, data was unavailable for some confounders (e.g., operative data, postprocedural echo data, and risk scores), which may also affect the results. Thirdly, the perioperative levels of other biomarkers, such as CK-MB, cardiac troponin T, and high-sensitivity troponin, were not collected for most patients in our center, but this complied with the standard practice for elective PCI. Thus, the prognostic values provided by cardiac biomarkers remains important for future research, and large prospective trials with more biomarker assessment and hard endpoints (e.g., cardiac death) are required.

## 6. Conclusions

In this observational study, we found the frequent occurrence of cTnI elevation in CAD patients who underwent complete PCI. Elevation in post-PCI cTnI ≥ 70× 99th percentile URL is independently associated with a high risk of MACEs during long-term follow-up for patients with TVD, but not for those with non-TVD. Elevation in post-PCI cTnI < 70× 99th percentile URL has no prognostic value regarding the risk of MACEs during follow-up for both TVD and non-TVD patients.

## Figures and Tables

**Table 1:** Characteristics of the study population by the presence or absence of TVD.

Variables	All (*n* = 1237)	Non-TVD Group (*n* = 798)	TVD Group (*n* = 439)	*p* Value
Male, *n* (%)	952 (77.0)	614 (77.0)	338 (77.0)	0.984
Age ± SD, years	58 ± 10	57 ± 10	59 ± 10	0.005
BMI (Q1,Q3), kg/m^2^	25.6 (23.7,27.7)	25.6 (23.6,27.7)	25.6 (23.9,27.8)	0.323
ACS, *n* (%)	868 (70.2)	557 (69.8)	311 (70.8)	0.750
Pre-PCI, *n* (%)	272 (22.0)	183 (23.0)	89 (20.3)	0.280
PAD, *n* (%)	7 (0.8)	2 (0.4)	5 (1.7)	0.117
Stroke, *n* (%)	51 (5.98)	30 (5.47)	21 (6. 9)	0.405
Hypertension, *n* (%)	787 (64.0)	489 (61.6)	298 (68.2)	0.021
Hyperlipidemia, *n* (%)	944 (76.7)	602 (75.9)	342 (78.1)	0.389
Diabetes mellitus, *n* (%)	350 (28.4)	202 (25.5)	148 (33.8)	0.002
Smoking history, *n* (%)	704 (57.2)	441 (55.5)	263 (60.2)	0.115
SBP (Q1,Q3), mmHg	120 (110,130)	120 (110,132)	120 (112,130)	0.496
LVEDD (Q1,Q3), mm	48 (45,50)	47 (45,50)	48 (45,50)	0.191
LVEF (Q1,Q3), %	65 (60,68)	65 (61,68)	64 (60,68)	0.058
TG (Q1,Q3), mmol/L	1.55 (1.13,2.14)	1.55 (1.10,2.14)	1.58 (1.16,2.20)	0.793
TC (Q1,Q3), mmol/L	4.03 (3.39,4.7)	4.02 (3.37,4.67)	4.04 (3.44,4.78)	0.283
HDL-C (Q1,Q3), mmol/L	1.02 (0.87,1.22)	1.02 (0.88,1.23)	1 (0.86,1.21)	0.302
LDL-C (Q1,Q3), mmol/L	2.31(1.80,2.92)	2.29 (1.77,2.88)	2.35 (1.87,2.99)	0.076
Lp(a) (Q1,Q3), mg/L	158.5 (68.18,353.23)	158.1 (63.23,337.25)	159.91 (73.68,363.56)	0.242
Hemoglobin ± SD, g/L	142.43 ± 14.49	143.09 ± 14.52	141.22 ± 14.37	0.031
NT-proBNP (Q1,Q3), pg/ml	561.5 (449.2,735.8)	555.8 (448.9,706.7)	579.75 (454.58,777.82)	0.049
Statins, *n* (%)	1214 (98.1)	782 (97.9)	432 (98.4)	0.515
Aspirin, *n* (%)	1233 (99.7)	792 (99.5)	438 (100)	0.336
Clopidogrel, *n* (%)	1224 (99.1)	787(98.9)	437 (99.5)	0.372
β-blockers, *n* (%)	1062 (85.9)	673 (84.4)	389 (88.6)	0.044
ACEI, *n* (%)	370 (30.0)	231 (29.0)	139 (31.7)	0.319
ARB, *n* (%)	342 (27.8)	208 (26.2)	134 (30.7)	0.089
cTnI < 1× URL, *n* (%)	499 (40.3)	342 (42.9)	157 (35.8)	0.055
cTnI 1–5× URL, *n* (%)	392 (31.7)	247 (31.0)	145 (33.0)	
cTnI 5–69× URL, *n* (%)	308 (24.9)	189 (23.7)	119 (27.1)	
cTnI ≥ 70× URL, *n* (%)	38 (3.1)	20 (2.5)	18 (4.1)	
MACE, *n* (%)	169 (13.7)	96 (12.0)	73 (16.6)	0.024
non-fatal MI, *n* (%)	24 (1.94)	14 (1.75)	10 (2.3)	0.523
non-fatal stroke, *n* (%)	34 (2.8)	21 (2.6)	13 (3.0)	0.734
unplanned revascularization, *n* (%)	86 (7.0)	49 (6.1)	37 (8.4)	0.130
re-hospitalization due to heart failure or severe arrhythmias, *n* (%)	5 (0.4)	4 (0.5)	1 (0.2)	0.797
all-cause death, *n* (%)	20 (1.6)	8 (1.0)	12 (2.7)	0.021

Notes: Data are expressed as mean + SD, median (Q1,Q3), or as number (%). TVD, three-vessel disease; BMI, body mass index; ACS, acute coronary syndrome; Pre-PCI, previous percutaneous coronary intervention; PAD, peripheral arterial disease; SBP, systolic blood pressure; LVEDD, left ventricular end diastolic diameter; LVEF, left ventricular ejection fraction; TG, triglyceride; TC, total cholesterol; HDL-C, high-density lipoprotein cholesterol; LDL-C, low-density lipoprotein cholesterol; Lp (a), lipoprotein (a); hs-CRP, hypersensitive C-reactive protein; NT-proBNP, N-terminal pro-B type natriuretic peptide; HbA1c, glycosylated hemoglobin A1 c; ACEI, Angiotensin converting enzyme inhibitors; ARB, Angiotension II receptor blockers; cTnI, cardiac troponin I; URL, upper reference limit; MACE, major adverse cardiovascular event.

**Table 2:** Univariate and multivariate predictors of 5-year MACE in the total cohort assessed by Cox regression analysis.

Variables	Univariate Analysis		Multivariate Analysis	
	HR (95%CI)	*p* Value	HR (95%CI)	*p* Value
Age	1.02 (1.01,1.04)	0.005		0.199
Male	0.91 (0.64,1.29)	0.592		0.267
SBP	1.002 (0.992,1.013)	0.627		0.767
LVEF	1.003 (0.981,1.025)	0.802		0.515
Hypertension	1.56 (1.11,2.19)	0.01	1.50 (1.01–2.20)	0.042
Diabetes mellitus	1.55 (1.14,2.12)	0.006		0.533
TVD	1.42 (1.04,1.92)	0.026	1.44 (1.03–2.02)	0.033
Hemoglobin	0.99 (0.98,1)	0.195		0.736
NT-proBNP	1 (1,1.001)	0.008		0.141
cTnI < 1× URL	Ref		Ref	
1–5× URL	0.80 (0.55–1.15)	0.227		0.406
5–69× URL	0.76 (0.51–1.14)	0.183		0.044
≥70× URL	2.09 (1.11–3.94)	0.022	2.47 (1.28–4.78)	0.007

Notes: SBP, systolic blood pressure; LVEF, left ventricular ejection fraction; TVD, three-vessel disease; cTnI, cardiac troponin I; URL, upper reference limit.

**Table 3:** Effect modification of TVD on the predictive value of cTnI for 5-year MACE.

	cTnI < 70× URL (<2.8 ng/mL)		cTnI ≥ 70× URL (≥2.8 ng/mL)		Unadjusted		*p* Value * for Interaction	Adjusted ^†^		*p* Value * for Interaction
	Total	Events (%)	Total	Events (%)	HR (95% CI)	*p* Value		HR (95% CI)	*p* Value	
Non-TVD	778	94 (12.1%)	20	2 (10.0%)	0.89 (0.23–3.60)	0.868	Ref	0.78 (0.19–3.20)	0.731	Ref
TVD	421	64 (15.2%)	18	9 (50.0%)	3.61 (1.80–7.27)	<0.001	0.08	4.43 (2.13–9.24)	<0.001	0.05

Notes: TVD, three-vessel disease; cTnI, cardiac troponin I; URL, upper reference limit; HR, hazard ratio; CI, confidence interval; * *p* value for the interaction test represents the interaction of cTnI level (≥70× URL vs. <70× URL) and participant group (Non-TVD vs. TVD) on MACE. ^†^ Adjusted for age, sex, systolic blood pressure, left ventricular end-diastolic volume, hypertension, diabetes mellitus, hemoglobin, and NT-proBNP. The cTnI <70× URL group is the reference group.

**Table 4:** Univariate and Multivariate Cox analysis of the predictive value of cTnI for 5-year MACE among TVD and non-TVD patients.

	Unadjusted HR (95% CI)	*p* Value	Adjusted HR * (95% CI)	*p* Value
TVD < 1× URL	Reference		Reference	
1–5× URL	0.64 (0.35–1.16)	0.139	0.61 (0.32–1.15)	0.126
5–69× URL	0.75 (0.41–1.36)	0.349	0.63 (0.33–1.20)	0.163
≥70× URL	2.91 (1.38–6.14)	0.005	3.32 (1.51–7.34)	0.003
Non-TVD < 1× URL	Reference		Reference	
1–5× URL	0.89 (0.56–1.41)	0.622	1.11 (0.67–1.84)	0.682
5–69× URL	0.73 (0.43–1.25)	0.250	0.58 (0.30–1.12)	0.106
≥70× URL	0.80 (0.19–3.29)	0.754	1.01 (0.24–4.32)	0.991

Notes: TVD, three-vessel disease; cTnI, cardiac troponin I; URL, upper reference limit; HR, hazard ratio; CI, confidence interval; * adjusted for age, sex, systolic blood pressure, left ventricular end-diastolic volume, hypertension, diabetes mellitus, hemoglobin, and NT-proBNP.

## Data Availability

Not applicable.

## Supplementary Materials

The following are available online at https://www.mdpi.com/article/10.3390/jcm11133896/s1, Table S1: Effect modification of TVD on the predictive value of cTnI for 5-year MACE in ACS patients, Table S2: The effect of TVD on the predictive value of cTnI for 5-year MACE in non-ACS patients, Table S3: Multivariate Cox analysis of the prognostic value of cTnI for 5-year MACE and single outcomes of the TVD and Non-TVD patients.
