# Supplementary material for: The Prognostic Value of Cardiac Troponin I in Patients with or without Three-Vessel Disease Undergoing Complete Percutaneous Coronary Intervention
Source: J Clin Med. 2022 Jul 4;11(13):3896. doi: 10.3390/jcm11133896 (PMC9267560; doi:10.3390/jcm11133896)
Supplement: Supplementary file 1 [file jcm-11-03896-s001.zip › jcm-1771254-supplementary.pdf]

Supplementary

Table S1. Effect modification of TVD on the predictive value of cTnI for 5-year MACE in ACS patients.

	cTnI<70×URL (<2.8 ng/ml)		cTnI⩾70× URL (⩾2.8 ng/ml)		Unadjusted		p Value * for Interaction	Adjusted†		p Value * for Interaction
	Total	Events (%)	Total	Events (%)	HR (95% CI)	p Value		HR (95% CI)	p Value	
Non-TVD	541	67(12.4%)	16	2(12.5%)	1.09 (0.27-4.44)	0.906	Ref	1.24 (0.29-5.32)	0.775	Ref
TVD	299	47(15.7%)	12	8(66.7%)	5.36 (2.52-11.37)	<0.001	0.05	5.30 (2.29-12.25)	<0.001	0.04

Notes: TVD, three-vessel disease; cTnI, cardiac troponin I; MACE, major adverse cardiovascular events; ACS, acute coronary syndrome; URL, upper reference limit; HR, hazard ratio; CI, confidence interval; *p value for interaction test represents interaction of cTnI level (≥70×URL vs. <70×URL) and participants groups (Non-TVD vs. TVD) on MACE. † Adjusted for age, sex, systolic blood pressure, left ventricular end-diastolic volume, hypertension, diabetes mellitus, hemoglobin, NT-proBNP. The cTnI<70×URL group is the reference group.

Table S2. The effect of TVD on the predictive value of cTnI for 5-year MACE in non-ACS patients.

	cTnI<70×URL (<2.8 ng/ml)		cTnI⩾70× URL (⩾2.8 ng/ml)		Unadjusted		Adjusted*	
	Total	Events (%)	Total	Events (%)	HR (95% CI)	p Value	HR (95% CI)	p Value
Non-TVD	237	27(11.4%)	4	0(0%)	-†	0.997	-†	0.997
TVD	122	17(13.9%)	6	1(16.7%)	0.98 (0.13-7.36)	0.983	0.99 (0.11-9.04)	0.990

Notes: TVD, three-vessel disease; cTnI, cardiac troponin I; MACE, major adverse cardiovascular events; ACS, acute coronary syndrome; URL, upper reference limit; HR, hazard ratio; CI, confidence interval; *Adjusted for age, sex, systolic blood pressure, left ventricular end-diastolic volume, hypertension, diabetes mellitus, hemoglobin, NT-proBNP. The cTnI<70×URL group is the reference group. † The number of events in non-TVD group with cTnI≥70×URL is 0 and cannot be analyzed.

Table S3. Multivariate Cox analysis of the prognostic value of cTnI for 5-year MACE and single outcomes of the TVD and Non-TVD patients.

Endpoints	TVD		Non-TVD	
	Adjusted HR* (95%CI)	P value	Adjusted HR* (95%CI)	P value
MACE				
<1×URL	Reference	-	Reference	-
1-5×URL	0.61 (0.32-1.15)	0.126	1.11 (0.67-1.84)	0.682
5-70×URL	0.63 (0.33-1.20)	0.163	0.58 (0.30-1.12)	0.106
≥70×URL	3.32 (1.51-7.34)	0.003	1.01 (0.24-4.32)	0.991
Non-fatal myocardial infarction				
<1×URL	Reference	-	Reference	-
1-5×URL	0.22 (0.02-1.91)	0.171	2.74 (0.88-8.58)	0.083
5-70×URL	0.55 (0.10-2.94)	0.485	0.39 (0.04-3.33)	0.386
≥70×URL	2.60 (0.27-24.70)	0.404	-†	-†
Non-fatal stroke				
<1×URL	Reference	-	Reference	-
1-5×URL	1.21(0.32-4.56)	0.784	1.56 (0.44-5.52)	0.494
5-70×URL	0.43 (0.07-2.62)	0.358	1.03 (0.24-4.40)	0.963
≥70×URL	-†	-†	5.21 (0.53-51.71)	0.158
Unplanned revascularization				
<1×URL	Reference	-	Reference	-
1-5×URL	0.59 (0.23-1.48)	0.257	0.68 (0.33-1.41)	0.303
5-70×URL	0.82 (0.35-1.94)	0.651	0.45 (0.18-1.12)	0.086
≥70×URL	4.94 (1.83-13.30)	0.002	0.72 (0.09-5.51)	0.750
Re-hospitalization due to heart failure or severe arrhythmias†				
All-cause death				
<1×URL	Reference	-	Reference	-
1-5×URL	0.74 (0.12-4.70)	0.749	2.98 (0.27-33.33)	0.375
5-70×URL	0.41 (0.06-2.71)	0.357	4.33 (0.36-51.33)	0.246
≥70×URL	9.04 (0.85-96.21)	0.068	-†	-†

Notes: TVD, three-vessel disease; cTnI, cardiac troponin I; URL, upper reference limit; HR, hazard ratio; CI, confidence interval; MACE, major adverse cardiovascular events.

* Adjusted for age, sex, systolic blood pressure, left ventricular end-diastolic volume, hypertension, diabetes mellitus, hemoglobin, NT-proBNP.

† Insufficient number of events for analysis.
